# Monitoring non-pharmaceutical public health interventions during the COVID-19 pandemic

**DOI:** 10.1038/s41597-021-01001-x

**Published:** 2021-08-24

**Authors:** Yannan Shen, Guido Powell, Iris Ganser, Qulu Zheng, Chris Grundy, Anya Okhmatovskaia, David L. Buckeridge

**Affiliations:** 1 McGill University, School of Population and Global Health, Department of Epidemiology, Biostatistics and Occupational Health, Montreal, H3A 1A2 Canada; 2 Johns Hopkins Bloomberg School of Public Health, Department of Epidemiology, Baltimore, 21205 USA; 3 London School of Hygiene and Tropical Medicine, Department of Epidemiology and Population Health, London, WC1E 7HT UK

**Keywords:** Health policy, Public health

## Abstract

Measuring and monitoring non-pharmaceutical interventions is important yet challenging due to the need to clearly define and encode non-pharmaceutical interventions, to collect geographically and socially representative data, and to accurately document the timing at which interventions are initiated and changed. These challenges highlight the importance of integrating and triangulating across multiple databases and the need to expand and fund the mandate for public health organizations to track interventions systematically.

In response to the COVID-19 pandemic, governments worldwide have implemented a range of measures to suppress or mitigate the spread of the virus. Given the lack of effective treatment and vaccine in the initial months of the pandemic, governments have relied on public health and social measures (PHSMs), also known as non-pharmaceutical interventions (NPI). Heterogeneous strategies have been used, implementing different interventions with variable stringency throughout the epidemic trajectory. This variation in public health practice is attributable in part to the limited evidence regarding the effectiveness of interventions to control COVID-19. This evidence gap is due in large part to challenges in measuring NPI.

The documentation and monitoring of NPIs, and public health interventions more generally, is not commonly conducted as part of public health surveillance or other routine monitoring activities. However, monitoring NPI would allow researchers and policymakers to (a) understand the progression of the epidemic, (b) quantify the effectiveness of interventions, (c) compare governmental policies in response to the pandemic across regions, and (d) analyze the effects of the COVID-19 pandemic on health, the economy and politics.

To address the need for measurement of public health interventions during the COVID-19 pandemic, multiple international research teams have established crowd-sourced, open databases that document NPI implemented worldwide. Many databases focus on a specific category of NPI such as travel restriction (e.g., https://www.wfp.org/emergencies/covid-19-pandemic and COVID Border Accountability Project (COBAP, https://covidborderaccountability.org/) or school closure (e.g., https://en.unesco.org/covid19/educationresponse). Some teams have built comprehensive trackers that record a wide spectrum of NPI launched by national and sub-national governments, such as the Oxford COVID-19 Government Response Tracker (OxCGRT, https://covidtracker.bsg.ox.ac.uk/)^1^ the Health Intervention Tracking for COVID-19 (HIT-COVID)^2^, the Complexity Science Hub COVID-19 Control Strategies List (CCCSL)^3^ project, the CoronaNet Research Project (CoronaNet)^4^ and the COVID-19 Government Measures Dataset from ACAPS (https://www.acaps.org) (Table 1). Some trackers collect data for a limited number of countries but with a high geographic resolution (e.g., a database of state-level social distancing responses in US^5^, a database of NPI in Brazilian municipalities^6^), while others maintain global coverage. Several teams have extended their work to collect economic policies and/or quantify the stringency of overall measures (e.g., OxCGRT, CoronaNet^4^, Response2covid19^7^ and COVID-19 Economic Stimulus Index^8^).

**Table 1:** Numeric summary of five NPI databases.

	OxCGRT	CoronaNet^4^	HIT-COVID^2^	ACAPS	CCCSL^3^
Number of records	29 153	48 816	11 999	20 987	6 500
Countries covered	187	201	140	193	57
Prop. of records at subnational level	0.40	0.54	0.60	0.14	0.33
Prop. of records in top 5 countries (prop. US)	0.32 (0.28)	0.36 (0.11)	0.46 (0.26)	0.09 (0.02)	0.32 (0.16)

Note: Summary reflects records in databases accessed on November 12, 2020.

Although these databases contain considerable information about the implementation of NPI worldwide, the quality of the information is influenced by challenges in measuring NPI. Here, we present three of the most important challenges for projects that use crowd-sourcing to capture a wide spectrum of non-pharmaceutical public health interventions worldwide. Given the rapid progress in this research, we do not intend to cover all existing projects. Rather, with reference to the five comprehensive global NPI databases listed in Table 1, we illustrate how these measurement challenges influence the information available about NPI implementation. We hope this commentary will support the informed use of NPI databases and contribute to the larger discussion of measuring and monitoring public health interventions.

## Challenge 1: Defining and Structuring What to Measure

At the moment, there is no standard vocabulary or widely accepted taxonomy for classifying NPI, which makes it challenging to consistently describe control measures for COVID-19. While vocabularies exist to describe clinical interventions, efforts to define and organize NPI in a similar fashion have been limited. Existing taxonomies of NPI, most notably, the World Health Organization’s (WHO) International Classification of Health Interventions (ICHI)^9^, lack coverage in certain areas and are not sufficiently expressive to describe NPI at the level of precision necessary to assess and compare their effectiveness. Another relevant terminology for describing NPI to control COVID-19 NPI is the WHO recommendations on uses of NPI for mitigating the risk and impact of epidemic and pandemic influenza^10^. However, these major classifications need to be updated to reflect the rapidly expanding knowledge about COVID-19 interventions.

In the absence of a single reference vocabulary, tracker teams have created different classification systems according to their needs, with definitions varying across trackers and each system evolving over the course of the pandemic. Many NPI, such as lockdowns, social distancing and border closures, seem self-explanatory, but they lack a clear and consistently understood definition. In different contexts, the same NPI can represent different actions, targeted mechanisms or stringency. For example, “lockdown” is often used ambiguously, referring at times to a cordon sanitaire, a curfew, a stay-at-home order, or to a sheltering of vulnerable populations. Some databases are accompanied by detailed descriptions of intervention terms in accessible documents, while other databases may only have cursory codebooks. There are also no clear mappings between the classes in the different systems, despite the overlap in the scope of NPI covered by the trackers. While it makes sense in each project to develop a taxonomy that best reflects the objectives of that project, incongruencies across tracker taxonomies impede attempts to compare and synthesize information across tracker databases.

Similarly to NPI classes, trackers inconsistently encode attributes of interventions, such as enforcement and targeted populations. Once an intervention is implemented, such attributes can be altered as the situation evolves. In fact, legislation and enforcement related to NPI have varied across levels of government and changed at an unprecedented pace. These dynamics are encoded to differing degrees across NPI databases. For example, different enforcement levels mean an intervention (e.g. mask-wearing) may be mandatory or only a recommendation by the government, reflecting meaningfully different realities on the ground. Furthermore, the target of intervention is crucial to encode. An intervention may be recorded broadly as flight restrictions in one database, while another database may record it as screening passengers from high-risk areas, stating the specific targeted population and action.

While documenting interventions with precision is essential for evaluating their relative effectiveness, it presents an additional challenge. A complex coding scheme required for such detailed documentation imposes significant additional burden on human analysts, both from the training and application perspective. The complexity of a vocabulary used to encode NPI can become a barrier to its adoption, especially in a time-sensitive context, like the COVID-19 pandemic. As a result, analysts may have to sacrifice granularity in favor of a simpler coding system.

Given these differences in definitions and encoding of information across trackers, researchers looking for data on interventions should define the NPI of interest and the important attributes of these interventions before searching for related data. With a clear scope and definitions, they can assess the extent to which available databases meet their needs in terms of types of interventions, targeted mechanisms and populations, and enforcement.

## Challenge 2: The Population and Countries to Track

Several NPI databases aim to provide global coverage, but the geographic coverage of the data may be uneven. This imbalance can result from limited time and resources as well as different priorities for each tracker project. Table 1 highlights the number of countries covered by each database, varying from 57 in the CCCSL database to 201 in CoronaNet. Among them, HIT-COVID primarily documents NPI implemented in African countries while CCCSL has focused its coverage on European countries.

The databases also vary in terms of the geographic resolution encoded in their records. While HIT-COVID (60%) and CoronaNet (54%) have many records of NPI at the sub-national-level (e.g., state, province, municipality), the majority of records in other databases are at the national level. We also noted inconsistencies across databases in the geographical level at which interventions were encoded. For example, measures such as national land border closures were often recorded as regional interventions, while measures such as regional school closures were often recorded as national measures. Moreover, interventions implemented for certain sub-populations, such as indigenous populations, may be underrepresented in the database.

Even for countries these databases cover, the recording of NPI may differ in completeness and detail from one country to the next. For example, HIT-COVID has the highest concentration of coverage in a small number of counties, with 46% of records from only 5 countries (26% being recorded for the US). The ACAPS database on the other hand more evenly covers the 193 countries in their database with only 9% of records from the top five countries. Records also varied geographically in terms of completeness for a specific NPI. For example, CoronaNet had 26 “External Border Restrictions” records for Germany over several months, while HIT-COVID only recorded 3 “Closed Border” measures on one single day. In Kenya, the two trackers respectively recorded 5 “External Border Restrictions” and 28 “Closed Border” measures over a similar period.

Given that geographic coverage and completeness vary across these databases, researchers should take account of this variation when selecting and using a tracker database. It may also be useful for tracker teams to focus on developing databases that target specific sub-populations or regions that have not otherwise been adequately covered.

## Challenge 3: The Timing of Interventions

**Fig. 1:**
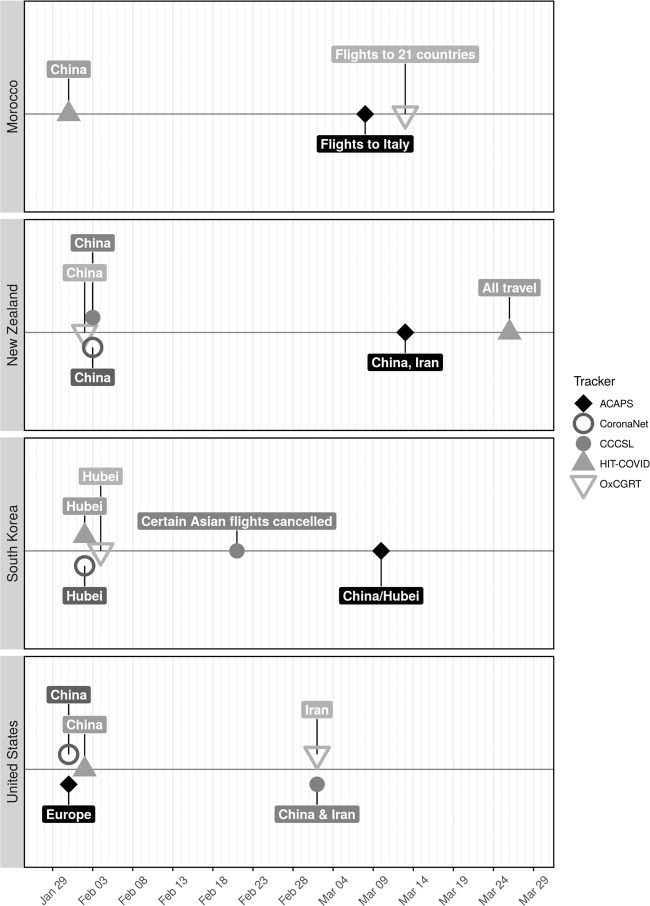
Comparison across five databases of the earliest recorded border restriction within four countries. Points represent dates at which measures are implemented. Each point is labelled with a note on the target population of border restrictions. The following measures were considered: “Ban arrivals from some regions” and “Ban on all regions or total border closure” (OxCGRT); “Closed border” (HIT-COVID); “Border restrictions” and “Airport restrictions” (CCCSL); “External border restrictions” (CoronaNet); “Border closure”, “Complete border closure”, “Visa restrictions”, and “International flights suspension” (ACAPS).

When NPI were implemented is an important measurement for many investigators who intend to use these databases. Despite a reasonable overlap in the categories of NPI recorded and the countries followed, the databases do not always agree on the date that an NPI was implemented within a country. For example, we compared a convenience sample of four countries (i.e., Morocco, New Zealand, South Korea and the US) according to five databases (Fig. 1). We used the first recorded international border restrictions (land, air, or water) for this comparison, because this measure is universally tracked, and, unlike more ambiguous measures such as social distancing, international border restrictions are relatively straightforward to map across databases. This analysis revealed widespread disagreement in the timing of measures, but also discrepancies in targets of border restrictions, such as which high-risk countries or travellers were banned from entry. Direct agreement was observed between only two trackers for bans on travellers from China entering New Zealand and a ban on Hubei travellers entering South Korea. In no other case did multiple databases record the first border measure on the same date, with the same target. We suspect that such disagreement in timing is more pronounced for measures whose definitions have more ambiguity, such as lockdown, where enforcement levels may also change over the progression of the pandemic.

The inconsistency in the timing of interventions across the databases may be due largely to the source documents. That is, both official and non-official documents do not consistently provide clear details about the date a measure was introduced. Similarly, online reports about NPI implementation can be overwritten by updated information without maintaining an archive of historical records. As a result, a volunteer may find an updated news or government report and code it as a new measure with a new start date. Although some NPI databases record “no change” or a similar status for ongoing interventions, inconsistencies in recorded NPI timing can still occur when the dates of NPI introduction and updating are not reported clearly. In these cases, the lack of clarity in the source documents and the crowd-sourced nature of the work can lead to confusion between the date when an intervention is implemented and the data when an intervention is noticed by volunteers, especially when the volunteers are not familiar with the language or the country of interest. More generally though, the inconsistency in the timing of interventions illustrates the multiple challenges in developing these databases. Some inconsistencies can be viewed as differences in definition or classification, with NPI that are thought to be equivalent across databases actually representing different interventions. In addition, the inconsistency may result from omissions, whereby databases have different scopes, objectives or priorities that lead to differential measurement for certain countries or interventions. Such differences can be amplified when more than one record is required to measure stages (e.g,, introduction, extending, expanding and phasing-out) of the same NPI over the course of the pandemic.

To ensure data quality, the teams who built these databases have adopted various approaches, such as standardizing data collection through procedures, tools, and volunteer training. Most trackers control for data quality by prioritizing government sources for their records. Many databases are also subject to validation processes after data entry, such as internal auditing in the HIT-COVID project, de-duplication and concordance checks for CCCSL, and inter-coder reliability assessment in the classification of NPI in CoronaNet. For investigators seeking data to support their studies, information about the quality control processes may be helpful in their selection of a database for a specific project. In some cases, additional validation using external sources may be indicated before using an NPI database.

## Future Use and Development of Crowd-Sourced NPI Trackers

In reviewing the current status of NPI databases, we have highlighted the importance of intervention definitions and measurement in terms of geography, population, and time. Clear definitions and accurate measurements are fundamental for any scientific endeavour^11^, including evaluating the effectiveness of NPI. When comparing impacts of interventions or strategies across countries or regions, bias may be introduced if the analysis does not account for the heterogeneity in terms of NPI timing, enforcement and targeted populations. Evaluation studies to quantify the validity and completeness of NPI implementation databases may be appropriate in some situations in order to allow adjustment for errors and missing data.

One approach to improving the quality and usability of data on NPI is to support collaboration between tracking efforts, through workshops and similar events (e.g., the international COVID-19 PHSMs Data Coverage Conference http://covid19-conference.org/) and by integrating multiple databases. The WHO has supported a project (WHO-PHSM^12^) to merge and harmonize data on COVID-19 public health and social measures, spearheaded by a team at the London School of Health and Tropical Medicine. Combining databases from seven international trackers, the WHO-PHSM database has de-duplicated, verified and standardized data into a unified taxonomy, geographic system and data format. In linking data and cataloguing evidence across trackers, the WHO-PHSM project has also identified which records represent different stages of the same NPI and provided multiple URLs and a summary of supporting documents for each record. Similarly, some projects have also adopted this synthesis approach and harmonized multiple datasets (e.g., CoronaNet^4^, Response2covid19^7^ and COVID-19 Economic Stimulus Index^8^). However, as with the underlying crowd-sourced databases of interventions, this synthesis work also entails considerable manual effort to verify and match taxonomies, among other tasks. This manual data processing slows the process of data dissemination and the “ground-up” nature of the overall approach produces synthesized databases that may still be missing data for geographic regions or sub-population not covered by any constituent database.

Another approach to integrating multiple databases is create an online directory of open databases tracking government policies. For example, the Oxford Supertracker (https://supertracker.spi.ox.ac.uk/) integrates 175 trackers into their platform and its scope goes beyond policies directly related to population health, including for example fiscal policies, research projects, and trade legislation. This curated approach enables researchers and policymakers to find original data sources that match their interests as well as multi-discipline collaboration. We support calls from Daly *et al*.^13^ for coordinated and gap-targeted data collection. High-income and English-speaking countries such as the US and the United Kingdom tend to have the largest number of NPI implementation records with detailed information, high geographic resolution and few missing values or errors. However, regions and sub-populations that are underrepresented in the databases, such as African countries and indigenous populations in high-income countries, are usually at greater risk of COVID-19 due to their lack of access to healthcare services and their elevated rates of other diseases.

Another concern is the potential deteriorating quality of the NPI databases as the pandemic continues and subsequent waves strike many regions worldwide. Many NPI have been reinforced or strengthened in response to the continuing pandemic, while other regions have remained in a state closer to normal. Additional challenges have been posed by the increasing granularity of NPI enforcement, emerging questions raised by various stakeholders, and discrepancies between NPI implementation and actual compliance. Meanwhile, volunteers may experience burnout and as financial support erodes, the sustainability of crowd-sourced tracking of NPI implementation is brought into question. Given the vast resources used to implement these interventions, which may have even larger economic and social effects, the evidence gap regarding their effectiveness is striking. If the current under-investment in monitoring public health interventions continues, it is conceivable that a similar effort to track NPI would need to be mounted for the future pandemics. An alternative would be to expand the mandate (and resources, especially financial support) for public health surveillance systems to include the systematic monitoring of NPI (and interventions more generally) given the fundamental importance of this type of data for understanding the effectiveness of public health efforts to control epidemics and more generally promote health.

A mandate to monitor interventions could be enabled through the use of novel data sources and emerging artificial intelligence (AI) technologies. Input from humans, especially experts in related domains, cannot be entirely replaced by AI. However, the motivation for adopting AI tools is to minimize the repetitive and laborious work so that human experts can concentrate on tasks that require their expertise. For example, machine translation can be used to overcome the language barriers, and web archiving and scraping tools can help collect and preserve dynamic online content automatically. Additionally, text classification tools can be used to filter NPI-related governmental documents and news reports, reducing the need for humans to comb through many documents, which may result in missing important details. In the CoronaNet project^4^, for example, text classifiers have been applied to identify news media reporting COVID-19 NPI policies and to classify the type of policy interventions described by the text. In another study^14^, a dynamic embedded topic model was applied to analyze online news from multiple sources and infer the timing and nature of NPI implementation. Despite these examples, the application of AI tools to these tasks is complicated and challenging. These challenges include the vast volume and noisy nature of online media from heterogeneous sources, the lack of high-quality labelled data, and the need to provide interpretable results. Further research and inter-disciplinary collaboration are therefore essential to realize the potential of AI methods to address these challenges.

## Conclusion

Incredible progress has been made through multiple efforts to establish databases that document the use of NPI to control the COVID-19 pandemic. While these databases can be valuable for research, their development is challenging in terms of defining NPI, identifying the population being monitored, and determining the timing of interventions. Different approaches to addressing these challenges can lead to omissions and inconsistencies when comparing databases from different tracking projects. These challenges highlight the need for researchers to consider integrating and triangulating multiple databases for a specific project. Given the importance of these data for understanding the effectiveness of public health efforts, we suggest that the mandate and funding of public health organizations be expanded to include the systematic tracking of interventions. This work could be supported by the use of artificial intelligence methods to reduce cumbersome manual work and speed data dissemination, but further research is necessary to realize this potential.
